# Disruption of Sphingolipid Biosynthesis Blocks Phagocytosis of *Candida albicans*

**DOI:** 10.1371/journal.ppat.1005188

**Published:** 2015-10-02

**Authors:** Fikadu G. Tafesse, Ali Rashidfarrokhi, Florian I. Schmidt, Elizaveta Freinkman, Stephanie Dougan, Michael Dougan, Alexandre Esteban, Takeshi Maruyama, Karin Strijbis, Hidde L. Ploegh

**Affiliations:** 1 Whitehead Institute for Biomedical Research and Massachusetts Institute of Technology, Cambridge, Massachusetts, United States of America; 2 Ragon Institute of MGH, MIT and Harvard, Cambridge, Massachusetts, United States of America; University of Melbourne, AUSTRALIA

## Abstract

The ability of phagocytes to clear pathogens is an essential attribute of the innate immune response. The role of signaling lipid molecules such as phosphoinositides is well established, but the role of membrane sphingolipids in phagocytosis is largely unknown. Using a genetic approach and small molecule inhibitors, we show that phagocytosis of *Candida albicans* requires an intact sphingolipid biosynthetic pathway. Blockade of serine-palmitoyltransferase (SPT) and ceramide synthase-enzymes involved in sphingolipid biosynthesis- by myriocin and fumonisin B1, respectively, impaired phagocytosis by phagocytes. We used CRISPR/Cas9-mediated genome editing to generate Sptlc2-deficient DC2.4 dendritic cells, which lack serine palmitoyl transferase activity. Sptlc2^-/-^ DC2.4 cells exhibited a stark defect in phagocytosis, were unable to bind fungal particles and failed to form a normal phagocytic cup to engulf *C*. *albicans*. Supplementing the growth media with GM1, the major ganglioside present at the cell surface, restored phagocytic activity of Sptlc2^-/-^ DC2.4 cells. While overall membrane trafficking and endocytic pathways remained functional, Sptlc2^-/-^ DC2.4 cells express reduced levels of the pattern recognition receptors Dectin-1 and TLR2 at the cell surface. Consistent with the *in vitro* data, compromised sphingolipid biosynthesis in mice sensitizes the animal to *C*. *albicans* infection. Sphingolipid biosynthesis is therefore critical for phagocytosis and *in vivo* clearance of *C*. *albicans*.

## Author Summary

The fungus *Candida albicans* is not only a commensal of the digestive system, but also a common cause of human opportunistic infections. Macrophages and dendritic cells can eliminate *C*. *albicans* by phagocytosis, a complex process that involves extensive membrane reorganization at the cell surface. The extent to which membrane lipids, including sphingolipids, contribute to the proper execution of phagocytosis remains largely unknown. Pharmacological blockade of sphingolipid biosynthesis by the small molecule inhibitors myriocin and fumonisin B1 impairs phagocytosis of *C*. *albicans*. DC2.4 dendritic cells genetically deficient in Sptlc2, the enzyme that catalyzes the first and rate-limiting step in the sphingolipid biosynthetic pathway, are likewise defective in phagocytosis of *C*. *albicans*. Sptlc2^-/-^ DC2.4 cells showed reduced binding of *C*. *albicans*, but overall membrane transport and protein secretion remained functional. Sptlc2-deficient cells express reduced levels of the receptors Dectin-1 and TLR2 at the cell surface, and are unable to form a normal phagocytic cup. Exogenous addition of the major ganglioside GM1 restored phagocytic ability of Sptlc2^-/-^ DC2.4 cells. Mice with compromised sphingolipid production upon *in vivo* treatment with fumonisin B1 fail to eradicate *C*. *albicans*, consistent with the *in vitro* results. Sphingolipids are thus essential for clearance of fungal infection through phagocytosis, and hence indispensable for the proper functioning of the innate immune system.

## Introduction

As a first line of defense against pathogens, the innate immune system relies on phagocytic cells that recognize and internalize foreign particulates. Phagocytosis of the fungal pathogen *Candida albicans* involves extensive membrane reorganization and actin remodeling at the plasma membrane for successful formation of a phagocytic cup [1–4]. Inevitably, the lateral movement of phagocytic receptors and other cofactors within the bilayer is influenced by the lipid composition of the membrane [5–8]. Nonetheless, the extent to which membrane lipids contribute to the proper operation of innate immune receptors remains largely unknown. Phosphoinositides, bioactive lipids localized mainly to the cytosolic leaflet of the plasma membrane, are essential during various stages of phagocytosis [9–14]. Formation of the phagocytic cup involves receptor clustering and cytoskeletal rearrangements at the site where the particle is initially bound. This step is highly coordinated and relies on modulation of phosphoinositide metabolism [9, 11].

Sphingolipids are conserved in all eukaryotes, and constitute 10–15% of total membrane lipids. They are heterogeneous in length, hydroxylation status and saturation of their acyl groups [15, 16]. Their distribution among the various biological organelles is distinct [16]. Sphingolipids are ubiquitous in the outer leaflet of the plasma membrane [17] where they are known to associate with cholesterol within the bilayer. Pathogens unavoidably interact with this class of lipids during phagocytosis. Evidence for the involvement of sphingolipids in fungal infections is mostly indirect, extrapolated from cholesterol depletion experiments [6], performed to explore the consequences of disrupting lipid rafts, which contain both cholesterol and sphingolipids. However, like many pharmacological interventions, the extraction of cholesterol using methyl-β-cyclodextrin is a relatively blunt instrument with inevitable off-target effects [18, 19].

Lipids are not template-encoded and are not uniquely confined to a given compartmentalized cellular organelle. This presents a challenge for the precise manipulation of their cellular levels and distribution. Consequently, it is difficult to distinguish between effects of altered lipid levels on the properties of a particular membrane or cellular compartment, and indirect effects caused by blocking steps upstream in biosynthetic or trafficking pathways. While this degree of complexity offers multiple points of attack for pharmacological and genetic intervention, manipulation of sphingolipid synthesis as a means of perturbing lipid homeostasis is comparatively underexplored. Studies of sphingolipid involvement in endocytosis of receptor-ligand complexes, or in phagocytosis of particulates such as microbes or opsonized red blood cells, has not yielded a consistent picture. Fumonisin B1 enhances phagocytosis of opsonized red blood cells, yet inhibits internalization of certain receptor-ligand combinations [20, 21]. We are not aware of any studies that examine the role of (glyco)sphingolipids in phagocytosis and clearance of pathogenic fungi such as *C*. *albicans*.

The biosynthetic pathway of sphingolipids and ceramides starts with the condensation of serine and palmitoyl CoA to yield 3-ketosphinganine in a reaction catalyzed by serine palmitoyl CoA transferase (SPT), a protein complex comprised of two subunits, Sptlc1 and Sptlc2 [22, 23]. Genetic ablation of either of the two subunits is embryonic lethal. Animals heterozygous for either the Sptlc1 or Sptlc2 null allele show reduced levels of sphingolipids [24]. A Chinese hamster ovary (CHO) cell line that lacks SPT failed to survive in the absence of exogenously added sphingoid base [25]. As to pharmacological approaches, SPT is inhibited by the small molecule myriocin [26]. Ceramide synthase accepts sphinganine and combines it with a fatty acyl CoA to yield dihydroceramide, a reaction that is blocked by the fungal metabolite fumonisin B1. As dihydroceramide is a precursor to all ceramides, application of fumonisin B1 has been used to manipulate ceramide and other sphingolipid levels, not only in tissue culture models but also in mice [20, 27]. Cellular sphingolipids are essential for the transport of viral glycoproteins from the Golgi apparatus to the cell surface [28, 29]. The role of sphingolipids in endocytosis of receptor-ligand complexes, or in phagocytosis of pathogens such as *C*. *albicans*, is largely unknown.

We used two approaches to manipulate sphingolipid biosynthesis. We used CRISPR/Cas9-mediated genome editing to generate dendritic cells deficient in Sptlc2. Such Sptlc2^-/-^ dendritic cells survive and grow. We applied the small molecule inhibitors fumonisin B1 and myriocin to interfere pharmacologically with sphingolipid synthesis. Both genetic and pharmacological blockade of sphingolipid synthesis caused a defect in phagocytosis of *C*. *albicans* by macrophages and dendritic cells. We thus find that sphingolipid biosynthesis is essential not only for efficient binding of particulates, but also for the formation of a normal phagocytic cup and subsequent internalization of the particulates. We show that sphingolipid biosynthesis is critical for cell surface expression of some pattern recognition receptors. Global membrane trafficking and endocytic pathways in Sptlc2-deficient cells are not overtly affected, at least for the parameters examined here. Mice treated with fumonisin B1 showed increased sensitivity to *C*. *albicans* infection *in vivo*, presumably due to a failure of phagocytic cells to properly engage the pathogen, thus leading to its uncontrolled extracellular proliferation. Therefore, our data show the importance of sphingolipids in phagocytosis and for the *in vivo* clearance of fungal infections.

## Results

### Inhibition of sphingolipid biosynthesis reduces phagocytosis of *C*. *albicans* by macrophages and dendritic cells

**Fig 1:**
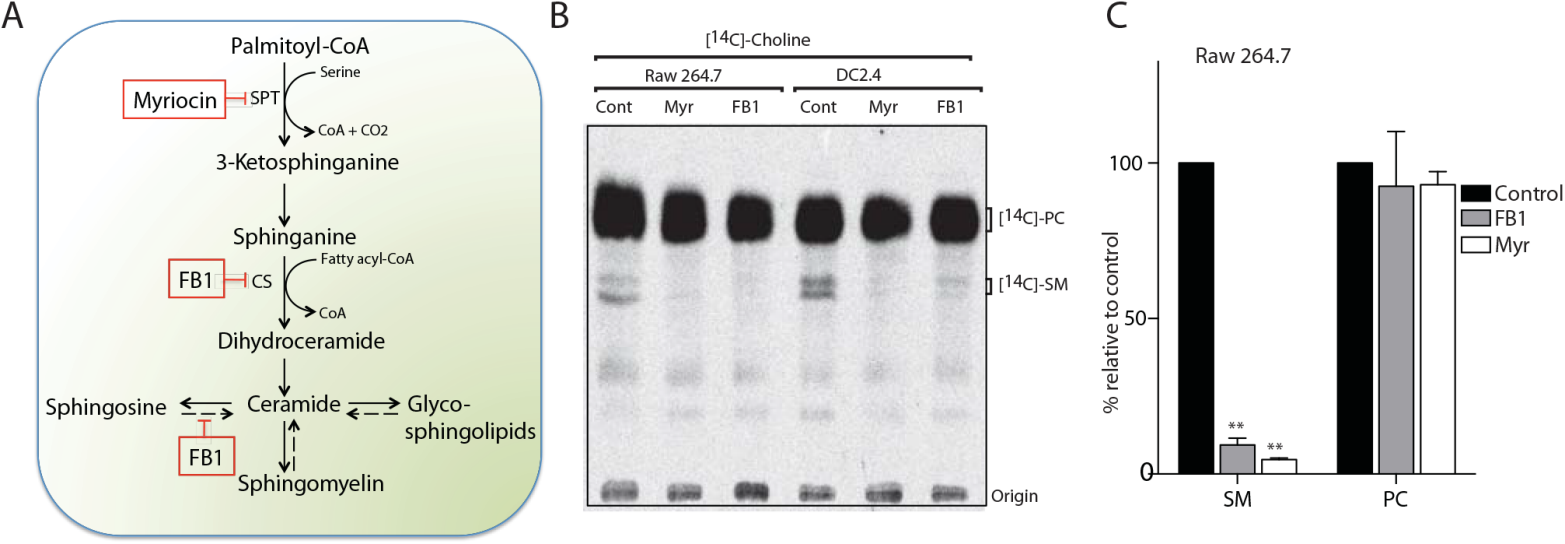
Myriocin and Fumonisin B1 block sphingolipid biosynthesis in RAW macrophages and dendritic cell lines. (A) Schematic representation of the sphingolipid biosynthetic pathway in mammalian cells. Myriocin, a serine palmitoyltransferase (SPT) inhibitor, and Fumonisin B1 (FB1), a ceramide synthase (CS) inhibitor, block sphingolipid biosynthesis (boxed). The salvage pathway is shown in broken arrows. (B) Myriocin- or FB1-treated cells were labeled with the sphingomyelin precursor *N*-methyl-[^14^C]-choline, and total lipids were extracted and analyzed by TLC and autoradiography. (C) Quantification of the [^14^C]-SM and [^14^C]-PC signals from [^14^C]-choline labeling experiment in B. Error bars display SD of three independent experiments. Unpaired t-test was used to analyze the significance of the observed differences. ** p < 0.001.

The small molecule inhibitors myriocin or fumonisin B1 (FB1) inhibit production of sphingolipids in mammalian cells. While myriocin blocks the activity of SPT-the first and rate-limiting reaction of this pathway- FB1 inhibits ceramide synthase [26, 30–32], causing a blockade in the production of ceramides, the backbone of all sphingolipids (Fig 1A).

To examine the role of sphingolipids in phagocytosis of *C*. *albicans*, we used myriocin and FB1 to manipulate sphingolipid levels in the macrophage cell line RAW264.7 and in the dendritic cell line DC2.4. Since the turnover of sphingolipids, especially that of sphingomyelin, is slow [33], cells were grown in the continuous presence of myriocin or FB1 for 4 days to obtain a significant reduction in sphingolipid levels. We observed no inhibition of cell growth in the presence of the inhibitors (S2 Fig). We then measured the biosynthesis of sphingomyelin, the major sphingolipid in mammalian cells. Cells were metabolically labeled with the sphingomyelin precursor *N*-methyl-[^14^C]-choline, total lipids were extracted and analyzed by TLC and autoradiography. Treatment of cells with myriocin or FB1 resulted in a significantly reduced level of [^14^C]-sphingomyelin (P < 0.0001; Fig 1B and 1C). However, the production of [^14^C]-phosphatidylcholine (PC) was not affected by either inhibitor (Fig 1B and 1C), in support of the selectivity of these inhibitors.

We next investigated the ability of RAW macrophages and DC2.4 cells treated with myriocin or FB1 to phagocytose *C*. *albicans*. We exposed inhibitor-treated RAW macrophages or DC2.4 cells to a blue fluorescent protein-expressing strain of *C*. *albicans* (*Candida*-BFP) for 60 and 90 minutes, and monitored phagocytosis by confocal microscopy. We used Alexa Flour 488-conjugated phalloidin to visualize the cells’ contours.

**Fig 2:**
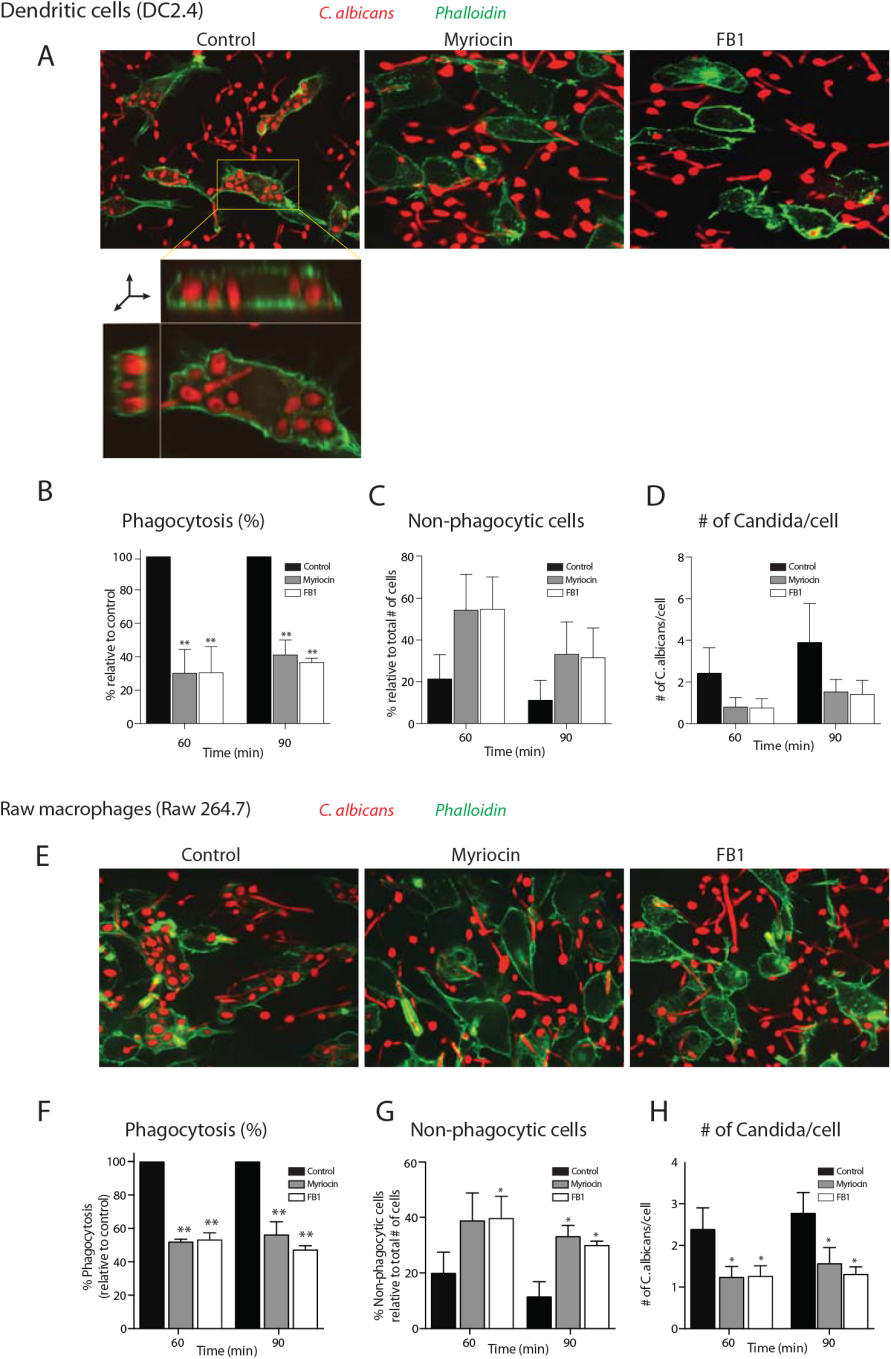
Inhibition of sphingolipid biosynthesis impaired the phagocytosis of *C*. *albicans*. (A) Confocal images of myriocin- or FB1-treated DC2.4 cells infected with C*andida*-BFP at multiplicity of infection (MOI) of 10. At 90 min, cells were fixed and stained with Alexa488-labeled phalloidin as a probe for filamentous actin to visualize the contours of the cells. XY plane of a Z stack as well as reconstructed XY and XZ planes are shown to demonstrate that the *C*. *albicans* are inside the cell (inset in first panel; for more information see S1 Movie). (B, C and D) Quantification of the number of internalized *Candida*-BFP (B), non-phagocytic cells (C) and the number of *Candida*-BFP per cell (D) of the experiments described in A. (E) Confocal images of myriocin- or FB1-treated RAW macrophages. Experiments were done as described in A. (F, G, H) Quantification of the number of internalized *Candida*-BFP (F), non-phagocytic cells (G), as well as the number of *Candida*-BFP per cell (H). Experiments were done as in A. The internalized *Candida*-BFP were quantified and are presented as the percentage relative to the control. Error bars display SD of three independent experiments where at least 200 cells were counted for each experiment. Unpaired t-test was used to analyze the significance of the observed differences. * p < 0.05; ** p < 0.001.

Cells treated with myriocin or FB1 showed significantly reduced levels of *C*. *albicans* phagocytosis compared to untreated cells (Fig 2A and 2E). DC2.4 cells treated with myriocin or FB1 showed 60–70% reduction (p < 0.001 for myriocin and p < 0.0001 for FB1), whereas RAW macrophages showed ~50% reduction (p < 0.0005 for both myriocin and FB1) at the 60 and 90 min time points (Fig 2B and 2F), with a corresponding increase in non-phagocytic cells (p < 0.05; Fig 2C and 2G). Of the cells that were able to internalize *C*. *albicans*, fewer organisms were internalized per cell (Fig 2D and 2H).

Next, we examined a time-course of myriocin treatment and investigated the cellular lipidome as well as phagocytosic ability. DC2.4 cells were treated with myriocin for 1, 2, 3 or 4 days and we then performed lipid profiling by LC-MS. The level of ceramide and cerebrosides were significantly reduced after 1 day of myriocin treatment (p<0.001; S1A, S1B and S1C Fig). The reduction in sphingomyelin was less pronounced and more gradual, showing the turnover of this lipid is slow [33]. DC2.4 cells treated with myriocin for 1 to 3 days showed a decrease in sphingomyelin of 35–40% (p < 0.05), while a ~50% reduction (p < 0.05) was found after 4 days of treatment (Fig 1C). We observed reduced levels of sphinganine (p < 0.001) after 4 days of myriocin treatment (S1D Fig). We noted a subtle increase in phosphatidylcholine in myriocin-treated DC2.4 cells (S1E Fig).

DC2.4 cells treated with myriocin for 1 or 2 days showed a 35–40% reduction in *C*. *albicans* phagocytosis (S1F Fig). A 3-day block in sphingolipid biosynthesis caused a ~50% reduction in phagocytosis (p < 0.001; S1E Fig) whereas we saw an even more pronounced reduction (60–70%; p<0.001) after 4 days (Fig 2B, see above). These data suggest that depletion to a certain level (for sphingomyelin) is essential to obtain a >60% blockade in phagocytosis.

### CRISPR/Cas9-mediated Sptlc2-knockout DC2.4 cells are defective in phagocytosis of *C*. *albicans*

**Fig 3:**
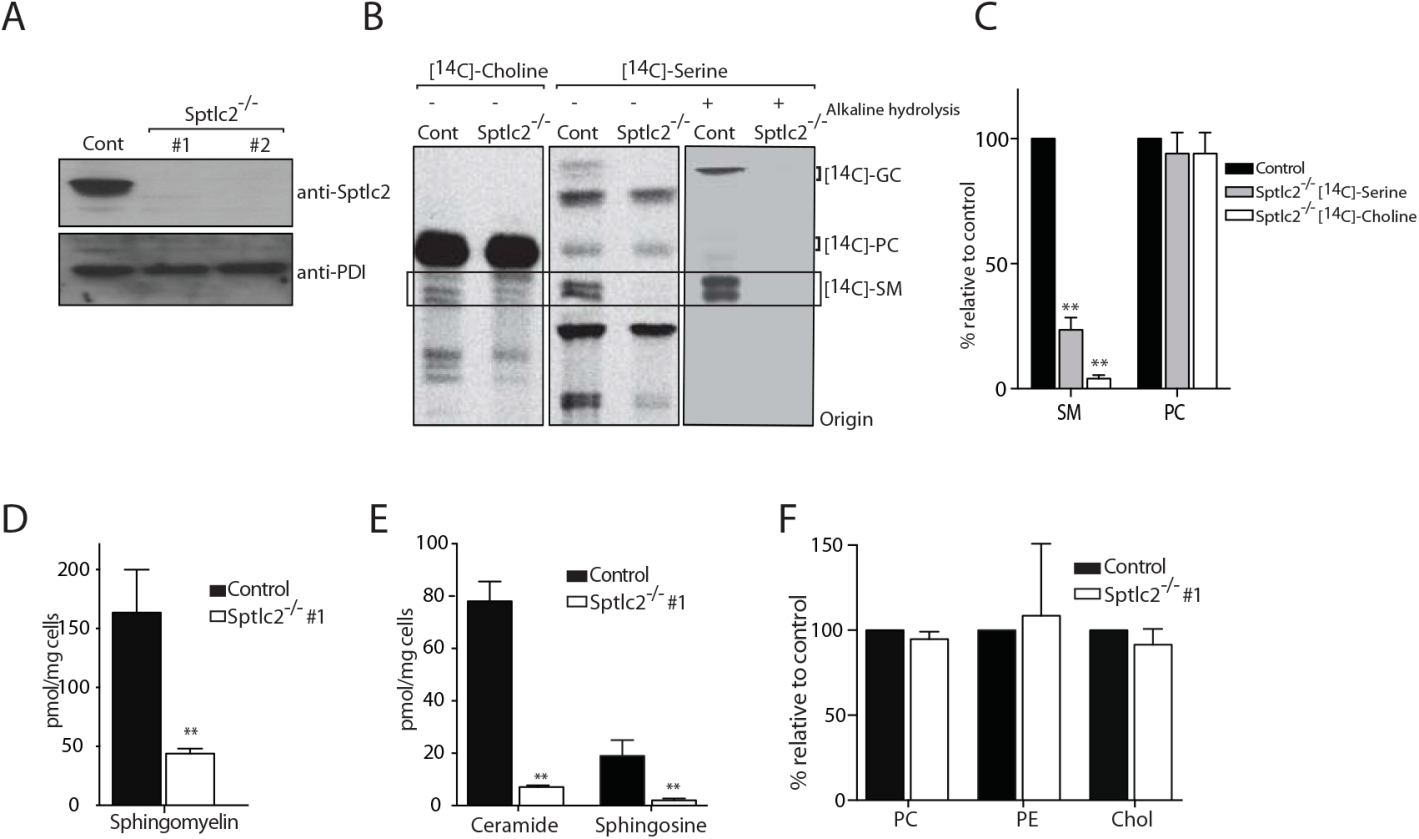
CRISPR/Cas9-mediated deletion of Sptlc2 in DC2.4 cells. (A) Immunoblot analysis of cell lysates from DC2.4 and two clonal isolates of Sptlc2^-/-^ DC2.4 cells using Sptlc2 antiserum. Protein disulfide isomerase (PDI) was used as a loading control. (B) Wild type DC2.4 and Sptlc2-deficient clonal isolates were labeled with *N*-methyl-[^14^C]-choline or *N*-methyl-[^14^C]-serine for 4 hours. Total lipids were extracted, and where indicated, glycerolipids were removed by mild alkaline hydrolysis. Lipids were then analyzed by TLC and autoradiography. (C) Quantification of the [^14^C]-SM and [^14^C]-PC signals from [^14^C]-choline and [^14^C]-serine labeling experiment in B. (D-F) Total lipids were extracted from control and Sptlc2^-/-^ cells and lipid profiling was performed using LC/MS. Levels of sphingomyelin (D), ceramide and sphingosine (E), and other phospholipids (PC and PE) and cholesterol (F) are shown. All graphs display SD of three independent experiments, and an unpaired t-test was used to analyze the significance of the data. ** p < 0.001. PC: phosphatidylcholine; PE: phosphatidylethanolamine.

To corroborate our observations that chemical inhibition of sphingolipid biosynthesis causes a defect in phagocytosis, and to exclude off-target effects of the drugs, we generated Sptlc2^-/-^ DC2.4 cell lines using CRISPR/Cas9 genome editing. Serine palmitoyl-CoA transferase (SPT), which catalyzes the first and rate-limiting reaction in the sphingolipid biosynthetic pathway, is an enzyme complex composed of two subunits, SPTLC1 and SPTLC2. We isolated two independent Sptlc2-deficient clones, for which the ablation of Sptlc2 was verified by immunoblotting of cell lysates (Fig 3A) using an anti-Sptlc2 serum.

To measure sphingolipid synthesis, we labeled cells either with *N*-methyl-[^14^C]-choline or with *N*-methyl-[^14^C]-serine. Sptlc2 transfers serine onto palmitoyl CoA to produce the first series of long-chain bases that eventually yields ceramides. Metabolic labeling with *N*-methyl-[^14^C]-serine showed a near-complete blockade of [^14^C]-sphingomyelin and [^14^C]-glucosylceramide production in Sptlc2-/- DC2.4 cells as demonstrated by thin layer chromatography (TLC) (Fig 3B and 3C). The level of [^14^C]-glucosylceramide, the major glycosphingolipid in mammalian cells, was below detection in Sptlc2-/- DC2.4 cells (Fig 3B). To confirm the identity of the sphingolipids as visualized by TLC, total lipid extracts were treated with mild alkaline sodium methoxide (Fig 3B) to hydrolyze glycerophospholipids and so reduce their contribution to the observed signal. Cells labeled with *N*-methyl-[^14^C]-choline also showed reduced levels of sphingomyelin compared to wild type cells (Fig 3B and 3C). Residual production of sphingolipids in *N*-methyl-[^14^C]-choline labeled cells likely results from a salvage pathway, in which ceramide is generated from sphingolipid turnover (Fig 1A; [34, 35]). Moreover, lipidomic profiling of Sptlc2^-/-^ DC2.4 cells by LC/MS showed that Sptlc2^-/-^ DC2.4 cells have significantly reduced levels of sphingosine (p <0.01), sphingomyelin (p < 0.001) and ceramide (p < 0.001) compared to control cells (Fig 3D and 3E). We also examined the level of other selected major classes of lipids, noting only a subtle increase in the level of phosphatidylethanolamine (PE; Fig 3F). No difference was seen for phosphatidylcholine (PC) or cholesterol levels in Sptlc2^-/-^ DC2.4 cells compared to controls (Fig 3F).

We found no obvious differences between Sptlc2^-/-^ DC2.4 and control cells with respect to cell division (S2B Fig) and morphology (S2A Fig). Acquisition of nutrients from the medium, disposal of waste products, or cytokinesis must therefore proceed in a manner consistent with survival and growth. We examined the morphology of Sptlc2^-/-^ DC2.4 cells by electron microscopy (EM). All organellar structures remain intact, and no obvious morphological differences were detected when comparing Sptlc2^-/-^ DC2.4 and controls (S2A Fig).

**Fig 4:**
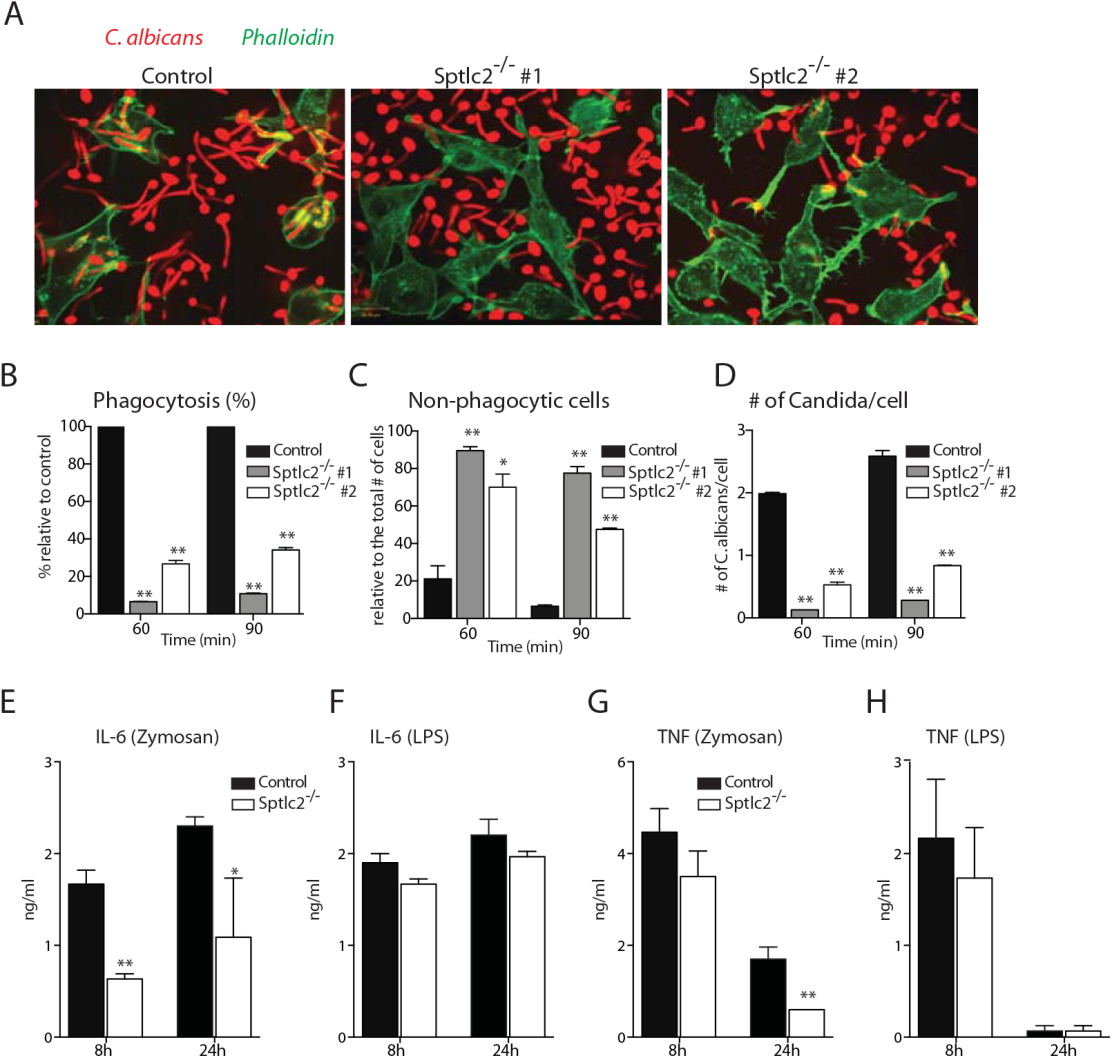
Sptlc2^-/-^ DC2.4 cells are defective in phagocytosis of *C*. *albicans* and produce reduced level of pro-inflammatory cytokines. (A) Confocal images of wild type and Sptlc2-deficient DC2.4 cells infected with C*andida*-BFP (at an MOI of 10). At 90 min post infection, cells were fixed and stained with phalloidin-Alexa488 to visualize the outline of the cells. (B-D) Quantification of the number of internalized *Candida*-BFP (B), non-phagocytic cells (C), and the number of *Candida*-BFP per cell (D). (E, H) Cells were treated with zymosan A (50 μg/ml) or LPS (1 μg/ml) for different time points and the level of IL-6 (E, F) and TNF-α (G, H) in the supernatant were determined by ELISA. All graphs display SD of three independent experiments, and at least 200 cells were counted for each experiment (B-D). Unpaired t-test was used to analyze the significance of the observed differences. * p < 0.05; ** p < 0.001.

To examine the phagocytic capacity of Sptlc2^-/-^ DC2.4 cells, we incubated them with *Candida*-BFP for 60 and 90 min, and scored phagocytosis by confocal microscopy. Sptlc2^-/-^ DC2.4 cells showed significantly less phagocytosis of *C*. *albicans* (p<0.0002) than wild type DC2.4 cells (Fig 4A and 4B) with a corresponding increase in non-phagocytic cells (Fig 4C). Similarly, the numbers of *C*. *albicans* per infected cell were significantly lower than in wild type DC2.4 cells (p < 0.0005; Fig 4D). Genetic ablation of Sptlc2 thus causes a defect in phagocytosis of *C*. *albicans*, underscoring the important role of an intact sphingolipid biosynthetic pathway for this process.

We next examined the impact of the Sptlc2 deficiency on phagocytosis of other particulates, such as fluorophore-conjugated zymosan or IgG-coated latex beads. We observed a blockade in phagocytosis of both zymosan and IgG-coated latex beads in Sptlc2^-/-^ DC2.4 cells (p < 0.001; S3 Fig). We tracked phagocytosis of zymosan particles by Sptlc2^-/-^ DC2.4 cells for more extended periods (4h and 6h post application), and still observed no internalization of zymosan (S3C Fig). Combined, our data show that Sptlc2^-/-^ DC2.4 cells are defective in internalization not only of *C*. *albicans*, but also of zymosan and IgG-coated beads.

### Sptlc2^-/-^ DC2.4 cells produce lower levels of pro-inflammatory cytokines

To investigate the role of sphingolipid biosynthesis in the production of cytokines upon fungal infection, we used zymosan to stimulate Sptlc2^-/-^ and control DC2.4 cells. Zymosan is a β-glucan-containing fungal particulate preparation that evokes inflammatory signals in macrophages and dendritic cells [36, 37]. Since pro-inflammatory cytokines are the main cytokines produced upon fungal infection [38], we measured production of IL-6 and TNF-α. In zymosan-stimulated cells, the production of IL-6 was reduced in Sptlc2^-/-^ DC2.4 cells compared to controls (Fig 4E). Similarly, we found a modest reduction in TNF-α levels in Sptlc2^-/-^ DC2.4 cells, and we obtained a significant difference after 24h of stimulation with zymosan (Fig 4G). We also determined the ability of Sptlc2^-/-^ DC2.4 cells to produce cytokines upon stimulation with the soluble ligand LPS. The production of IL-6 and TNF-α was not significantly affected in Sptlc2-deficient cells in response to LPS stimulation (Fig 4F and 4H).

### FB1 treated mice show increased sensitivity to *C*. *albicans* infection

We next investigated the role of sphingolipid biosynthesis in *C*. *albicans* infection *in vivo*. Because Sptlc1 and/or Sptlc2 knockout mice are embryonic lethal [24, 39], we used FB1 to inhibit sphingolipid biosynthesis in mice. Unlike FB1, myriocin is known to exhibit an immunosuppressant property *in vivo* [26, 40] and therefore we considered it not compatible for our *in vivo* study. We first established the dose and duration of FB1 treatment required to obtain reduced levels of sphingolipids without compromising the health of the treated mice. Mice received daily subcutaneous injections of 2 mg/kg FB1. Although FB1 also inhibits sphingolipid production in *C*. *albicans* [41], we used a far lower concentration in mice than that required for use in *C*. *albicans*. The concentration of FB1 used in mice does not affect growth of *C*. *albicans* (S4B Fig).

**Fig 5:**
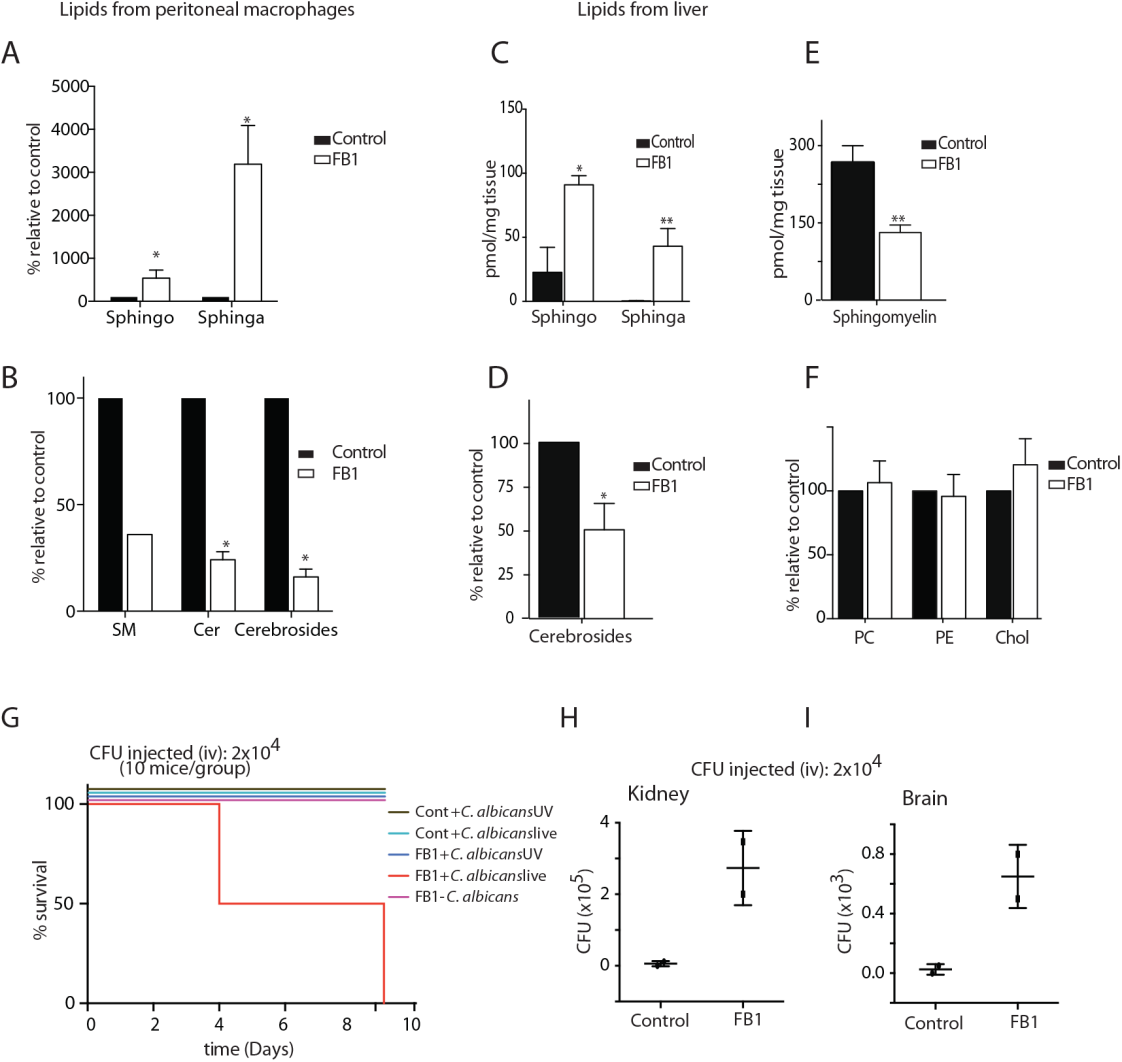
treated with FB1 show increased susceptibility to *C*. *albicans* infection. (A—F) Total lipids were extracted from control and FB1-treated mice and subjected to LC/MS lipid profiling. The different classes of lipids from peritoneal macrophages (A, B) and liver tissue (C—F) are shown. (G) Survival curve of control and FB1-treated mice infected with live or UV-killed *C*. *albicans*. (H, I) Fungal load was determined from the kidney and brain tissues collected from the control and FB1-treated mice that were infected with *C*. *albicans*, and the number of colony forming units (CFU) is given. SM, sphingomyelin; Cer, Ceramide; Sphingo, Sphingosine; Sphinga, Sphinganine; Chol, Cholesterol.

Mice treated with FB1 showed no gross differences in health or behavior compared to untreated animals (S4A Fig). After 5 days, mice were sacrificed and lipidomic analysis was performed by LC/MS on lipids extracted from peritoneal macrophages and liver tissue. As expected, the level of sphinganine in FB1-treated mice increased > 30-fold and > 40-fold in peritoneal macrophages (Fig 5A) and liver (Fig 5C), respectively. Sphingosine increased 3 to 4-fold in both peritoneal macrophages (Fig 5A) and liver tissue (Fig 5C). Consistent with these data, FB1-treated mice showed reduced levels of ceramide, sphingomyelin, and cerebrosides (glucosylceramide and galactosylceramide) in both peritoneal macrophages (Fig 5B) and liver (Fig 5E and 5D). We observed no differences in the levels of total cholesterol and the phospholipids PE and PC (Fig 5F), indicating specificity of the FB1-imposed blockade.

Mice treated with FB1 for 5 consecutive days received 2x10^4^ colony forming units (CFU) of live or the UV-killed equivalent of *C*. *albicans* via tail vein injection. Mice continued to receive FB1 treatment daily and were closely monitored for survival and overall well-being. FB1-treated mice were highly susceptible to infection with live *C*. *albicans*, with no animals surviving beyond 9 days post infection, whereas at this dose of *C*. *albicans*, all of the untreated but infected animals survived (Fig 5G). FB1-treated mice injected with UV-killed *C*. *albicans* showed no obvious signs of ill health compared to control mice.

We examined the *C*. *albicans* load in kidney and brain of infected animals on day 9. In FB1-treated mice, both organs were heavily colonized with *C*. *albicans* (Fig 5H and 5I). Fungal load was also determined on day 5. We therefore concluded that the cause of death in FB1-treated mice infected with live *C*. *albicans* was systemic candidiasis, rather than septic shock resulting from intravenous delivery of ligands for TLRs or other pattern recognition receptors.

### Sptlc2^-/-^ DC2.4 cells are defective in binding of particulates during phagocytosis

**Fig 6:**
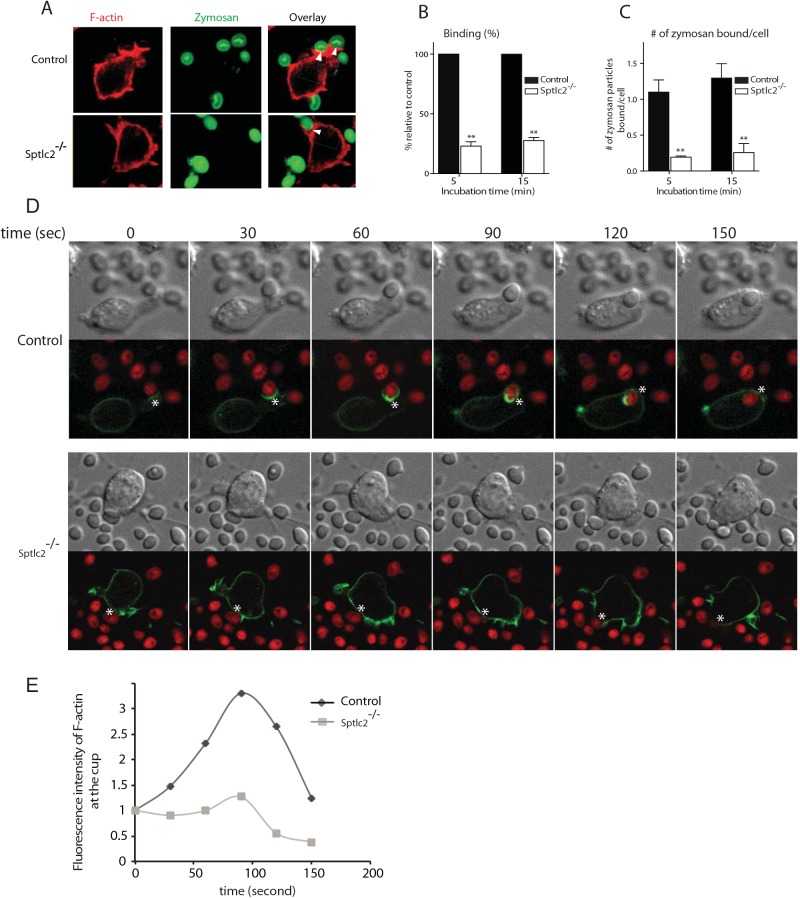
Sptlc2^-/-^ DC2.4 cells are defective in particulate binding and phagocytic cup formation. (A) Control and Sptlc2^-/-^ DC2.4 cells were incubated with Alexa fluor 647-conjugated zymosan and their ability of binding the particulates was examined by confocal microscopy. Arrows indicate sites of binding. (B, C) Quantification of the number of bound zymosan particles (B) and the number of zymosan particles bound per cell (C). The bound particles were quantified and presented as the percentage relative to the control. (D) Control and Sptlc2^-/-^ DC2.4 cells stably expressing LifeAct-mCherry (F-actin) were incubated with *Candida*-BFP (shown in red) and imaged using confocal microscopy. Images captured at 30-second intervals are shown. A representative of three independent experiments is shown. (E) A graph showing a quantitation of F-actin fluorescence intensity in the course of formation of a phagocytic cup (shown in asterisks), images were taken as described in D. All graphs display SD of three independent experiments, and an unpaired t-test was used to analyze the significance of the observed differences. ** p < 0.001.

To determine the step at which sphingolipids are required during phagocytosis, we used confocal microscopy and examined the ability of Sptlc2-/- DC2.4 cells to bind zymosan-Alexa647. Alexa fluor 647-conjugated zymosan was added to cells on ice (10 zymosan particles per cell), which were then transferred to 37°C for 5 and 15 min. Sptlc2-/- DC2.4 cells showed significantly less binding of zymosan than control DC2.4 cells, both at 5 and 15 min of incubation (p = 0.0008; Fig 6A and 6B). The number of zymosan particles bound per cell was significantly higher for control cells than for Sptlc2^-/-^ DC2.4 cells (p < 0.0001; Fig 6C). Together, these results underscore the role of an intact sphingolipid biosynthetic pathway in binding particulates, which might involve any of a number of different surface structures.

### Sptlc2^-/-^ DC2.4 cells are defective in forming a typical phagocytic cup

Why are Sptlc2^-/-^ DC2.4 cells defective in phagocytosis? Phagocytosis involves remodeling of the actin cytoskeleton to guide and shape the membrane around the pathogen to form a phagocytic cup [42, 43]. We investigated actin-driven phagocytic cup formation in Sptlc2^-/-^ DC2.4 cells. We generated Sptlc2^-/-^ DC2.4 and wild type DC2.4 cell lines that stably express mCherry-tagged LifeAct, a biosensor that visualizes the distribution of filamentous actin in living cells [44]. After incubating the respective cell lines with *Candida*-BFP, we performed live cell imaging/confocal microscopy to capture phagocytic events. Wild type DC2.4 cells remodeled their actin cytoskeleton as expected, with clear evidence of recruitment and polymerization of actin in the course of phagocytic cup formation, and its subsequent depolymerization upon formation of a phagosome (Fig 6D and 6E; S1 and S2 Movies). Accordingly, actin continues to concentrate at the base of the cup where cells contact *C*. *albicans* and remains there until closure of the phagosome. Subsequently, actin concentrations decrease at the phagocytic cup, creating a belt-shaped band of actin that stretches outwards for the successful engulfument of the *C*. *albicans* particle (Fig 6D; Fig 9). For Sptlc2^-/-^ DC2.4 cells, even though random actin polymerization events occur at the cell surface, we only rarely observed the successful completion of a typical phagocytic cup (Fig 6; S2 and S3 Movies).

### Sptlc2^-/-^ DC2.4 cells express reduced level of pattern recognition receptors at the cell surface

Phagocytosis is not only an actin-driven cellular activity, but is also a receptor-mediated process initiated upon recognition of particulates by pattern recognition receptors (PRRs) expressed at the cell surface of phagocytes [45]. The PRRs enable immune cells to discern pathogen-associated molecular patterns (PAMPs) found on the cell wall of most microbial pathogens [46].

**Fig 7:**
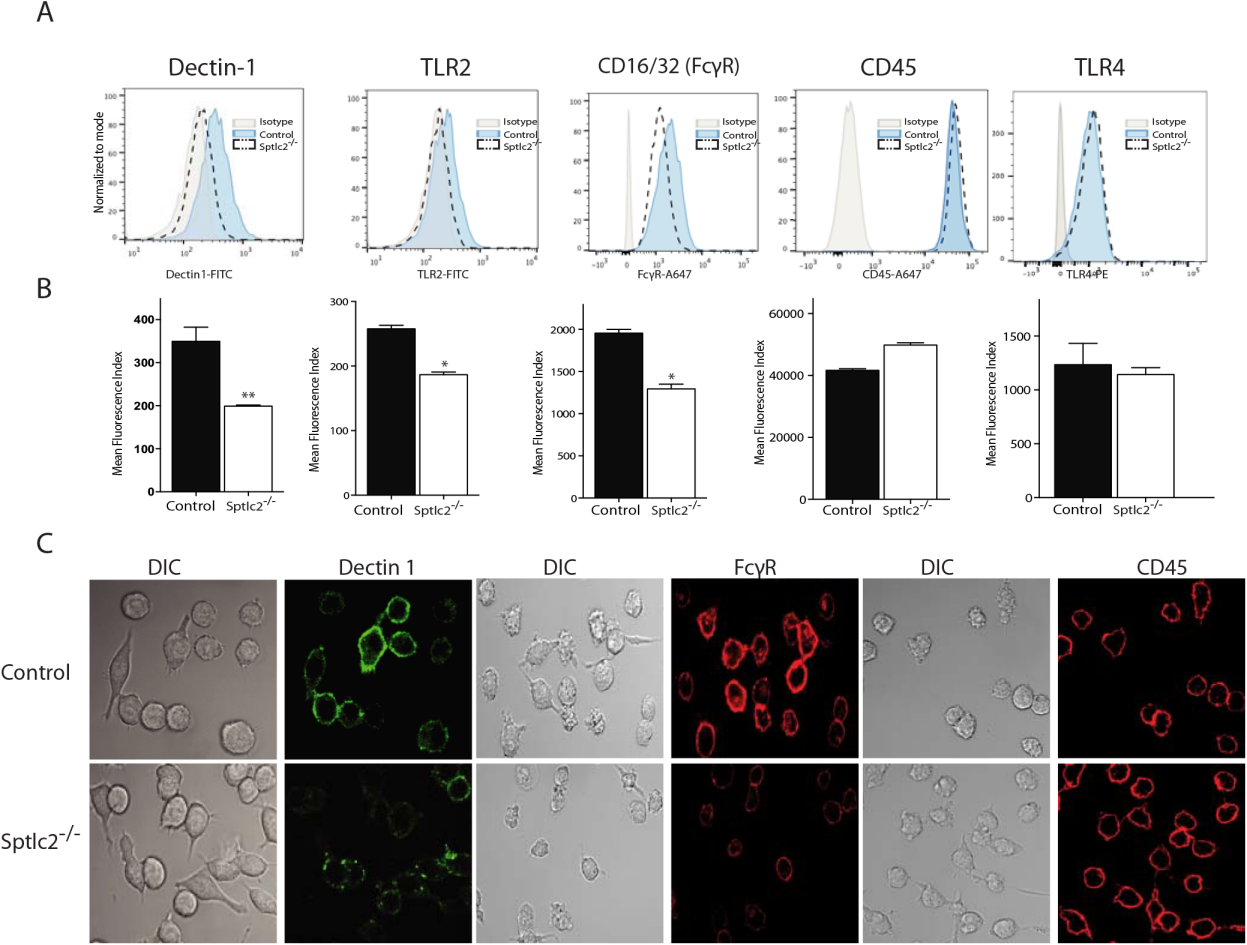
Sptlc2^-/-^ cells express reduced level of pattern recognition receptors at the cell surface. (A) Flow cytometry of dectin-1, TLR2, CD16/32 (FcγR), CD45 and TLR4 at the cell surface of Sptlc2^-/-^ and control DC2.4 cells. (B) Graphs showing quantification of cell surface expression of the receptors described in A. (C) Confocal images of the control and Sptlc2^-/-^ DC2.4 cells incubated with antibodies specific for Dectin 1, FcγR or CD45 on ice for 30 min.

Are sphingolipids essential for cell surface disposition of PRRs? We examined PPR expression on Sptlc2^-/-^ DC2.4 and control cells by flow cytometry and confocal microscopy. Surface expression of Dectin-1, TLR2, and FcγR was reduced in Sptlc2^-/-^ DC2.4 cells compared to control cells (Fig 7A–7C). Cell surface expression of TLR4, a toll-like receptor that recognizes lipopolysaccharides, was not affected (Fig 7A and 7B).

Similarly, surface expression of CD45, a receptor-linked protein tyrosine phosphatase, was not affected in Sptlc2^-/-^ DC2.4 cells (Fig 7A, 7B and 7C), showing at least some degree of specificity for the role of sphingolipids in surface disposition of PRRs. Despite repeated attempts to measure surface expression of Galectin-3, DC-SIGN and mannose receptor CD206, we were unable to detect these proteins in DC2.4 cell lines by flow cytometry or confocal microscopy. It is possible that the DC2.4 cell line and its derivatives express only very low levels, or are negative for these markers.

### Sptlc2^-/-^ DC2.4 cells do not show a generic defect in membrane trafficking

**Fig 8:**
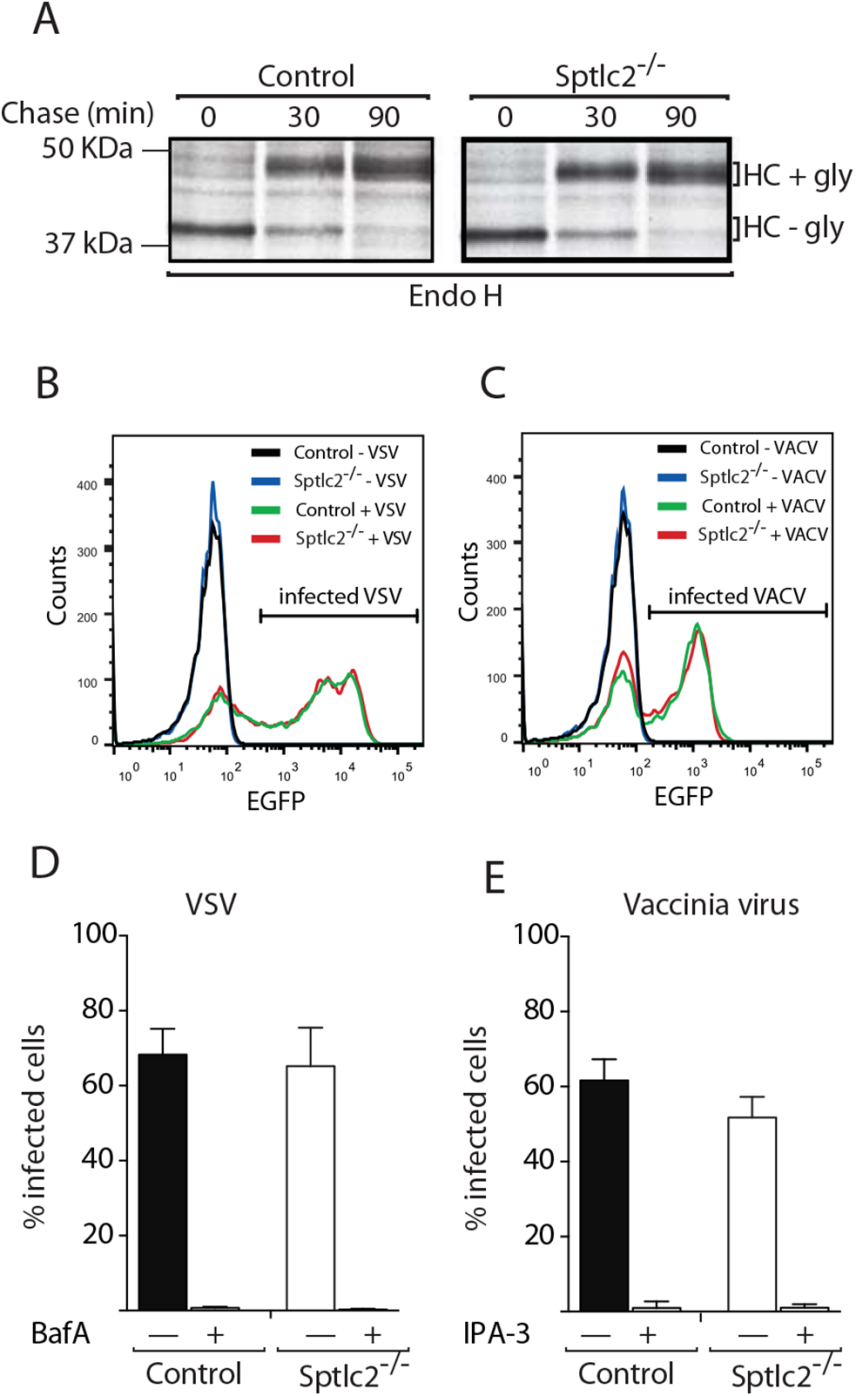
Sptlc2-/- DC2.4 cells do not show a generic defect in membrane trafficking and can be infected with endocytosis-dependent viruses. (A) Wild type and Sptlc2^-/-^ DC2.4 cells were pulse-labeled with [^35^S]-methionine/cysteine for 20 min and chased for different time points. Cells were lysed and MHC class I heavy chain [47] molecules were immunoprecipitated, treated with Endo H, and analyzed by SDS/PAGE and autoradiography. (B and C) Cells were infected with VACV WR E eGFP or VSV eGFP [48]. At 6 h post infection, cells were harvested, and infected cells quantified by flow cytometry. (D and E) Quantification of experiments performed as described in B and C, respectively. Where indicated, cells were pretreated with 50 μM 3-indolepropionic acid (IPA-3), 30 nM bafilomycin A1 (BafA), or 0.1% DMSO and kept in the presence of the drugs throughout the experiment. Representative histograms (B and C) and mean values ± SD from three independent experiments (D and E) are displayed.

We determined whether Sptlc2-/- DC2.4 cells display normal overall membrane trafficking by exploring overall secretion, and the synthesis and maturation of Class I MHC products, a type-I membrane protein expressed on all nucleated cells. Biosynthetic incorporation of [^35^S]methionine/cysteine in control and Sptlc2^-/-^ DC2.4 cells was comparable (S5B Fig). We monitored levels and composition of secreted proteins in Sptlc2^-/-^ DC2.4 cells and control cells. Cells were labeled with [^35^S]-methionine/cysteine for 30 min and chased for different times. Secreted proteins were analyzed by SDS/PAGE and autoradiography, using cells maintained at 4°C as controls. Sptlc2-deficient and wild type cells secreted comparable amounts of protein (S5A Fig). We also monitored the synthesis and maturation of Class I major histocompatibility complex (MHC) products by pulse-chase analysis. Sptlc2^-/-^ DC2.4 cells transport class I MHC HC at rates equal to control cells, as assessed by rate and extent of acquisition of Endo H resistance (Fig 8A).

**Fig 9:**
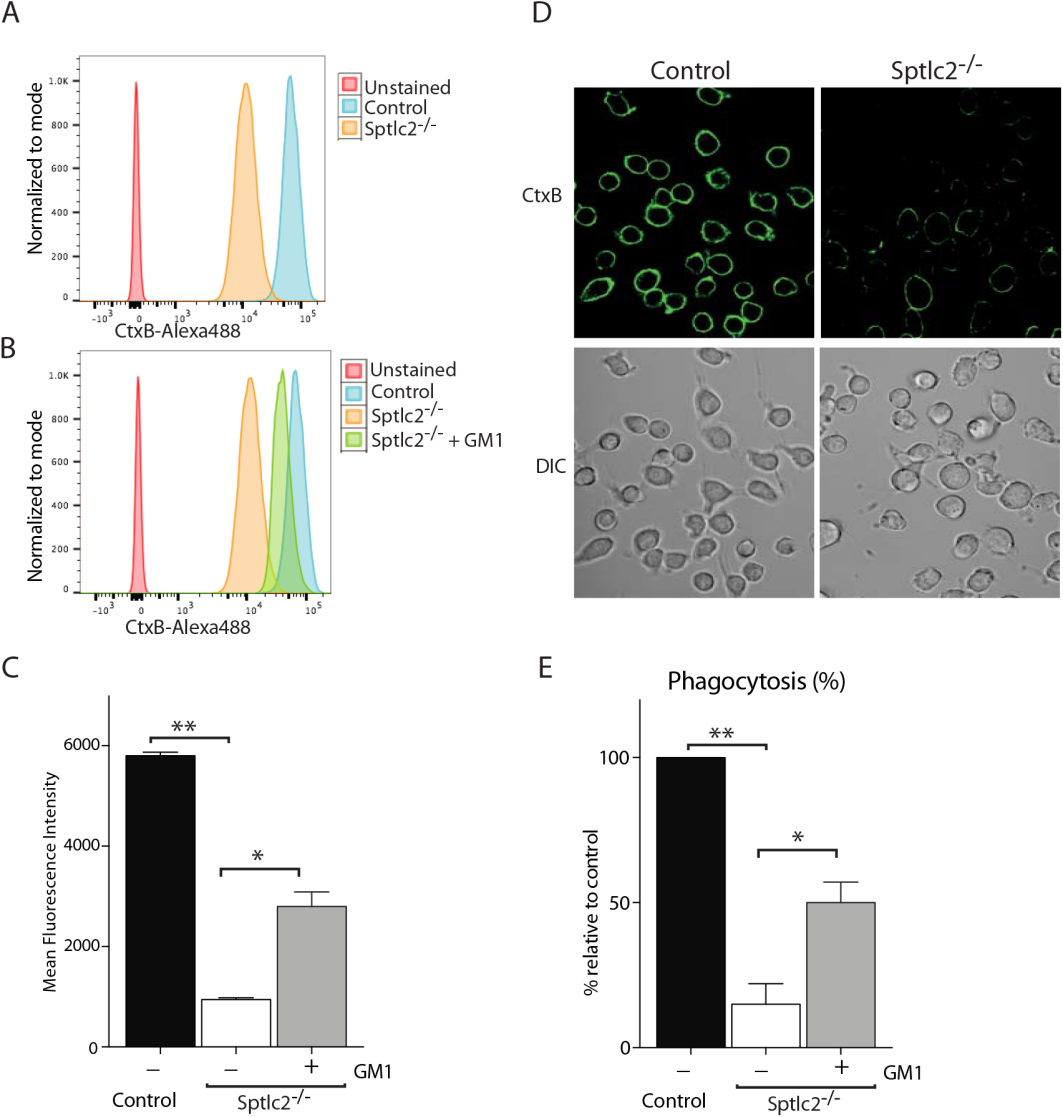
Exogenous addition of GM1 restores the phagocytic ability of Sptlc2^-/-^ DC2.4 cells. [13] Flow cytometry of Sptlc2^-/-^, Sptlc2^-/-^ + GM1 and control DC2.4 cells stained with CtxB. (C) Graphs showing quantification of cell surface staining of CtxB described in A and B. (D) Confocal images of the control and Sptlc2^-/-^ DC2.4 cells incubated with CtxB-Alexa488 on ice for 30 min. (E) Control, Sptlc2^-/-^ and Sptlc2^-/-^ + GM1 DC2.4 cells were incubated with *Candida*-BFP and their ability to phagocytose the fungus was examined by confocal microscopy. The internalized *Candida*-BFP were quantified and presented as the percentage relative to the control. All graphs display SD of three independent experiments, and an unpaired t-test was used to analyze the significance of the data. ** p < 0.001, * p < 0.05.

### Sptlc2^-/-^ DC2.4 cells can be infected with endocytosis-dependent viruses

To verify that endocytic pathways were functional in Sptlc2^-/-^ DC2.4 cells, we tested whether Sptlc2^-/-^ cells could be infected with two viruses known to rely on distinct routes of endocytosis for host cell entry. Mature virions (MVs) of vaccinia virus (VACV), the prototypic poxvirus, enter cells by virus-induced macropinocytosis [49]. In contrast, vesicular stomatitis virus (VSV), a rhabdovirus, employs clathrin-mediated endocytosis and acid-mediated fusion from early endosomes to infect host cells [50]. Equal numbers of wild type and Sptlc2^-/-^ DC2.4 cells were infected with-expressing viruses. Both virus strains encode eGFP as a non-structural protein. Successful transcription and translation require delivery of the viral genomes to the host cell cytosol. The fraction of infected cells was determined 6 h after infection by measuring the levels of eGFP using flow cytometry (Fig 8B and 8C). VACV infection was robust in Sptlc2^-/-^ cells, although the number of infected cells was moderately reduced compared to wild type cells (Fig 8D). To verify that VACV infection in wild type and Sptlc2^-/-^ DC2.4 cells indeed relied on macropinocytosis, infection experiments were also performed in the presence of 3-indolepropionic acid (IPA-3), an inhibitor of p21-activated kinase 1 (PAK1) known to be required for macropinocytosis [48]. VACV infection was completely abrogated by IPA-3 in both wild type and knockout cells, suggesting that the infectious entry mechanism of VACV in DC2.4 cells indeed relies on macropinocytosis (Fig 8E). The fraction of VSV-infected cells was nearly identical in wild type and knockout cells (Fig 8B and 8D). Moreover, infection was sensitive to bafilomycin A1, an inhibitor of endosomal acidification (Fig 8D). Infection of DC2.4 cells with VSV indeed required acidified endosomes and endocytosis. We observed normal infectivity of VSV in Sptlc2^-/-^ DC2.4 cells. Collectively, the use of VACV and VSV as well-defined endosomal cargo confirmed that endocytic pathways were functional in Sptlc2^-/-^ DC2.4 cells, consistent also with robust growth of Sptlc2^-/-^ DC2.4 cells in tissue culture, which depends in part on endocytic uptake of nutrients.

### Exogenous addition of GM1 partially restores phagocytosis defect in Sptlc2^-/-^ cells

Since we were unable to determine the level of gangliosides in our lipidomic analysis using LC-MS, we used a fluorescently labeled cholera toxin subunit B (CtxB). CtxB binds to GM1, a ganglioside that contains a sialic acid residue conjugated to a ceramide moiety. CtxB is commonly used as a tool to visualize lipid microdomains at the cell surface [51]. Both confocal microscopy and flow cytometry showed a significantly reduced level of CtxB binding (~85%) in Sptlc2^-/-^ DC2.4 cells compared to the control (Fig 9A and 9D). This is consistent with our lipidomic analysis where we saw a decrease in ceramide, the backbone of all gangliosides including GM1 (Fig 3C). Membrane organization in Sptlc2^-/-^ DC2.4 cells must therefore be different to account for the defects in phagocytosis, while still compatible with other membrane-associated phenomena, such as the operation of the secretory pathway, endocytic entry of viruses and cell growth more generally, as described above.

Next, we assessed whether addition of GM1 exogenously could restore the defect in phagocytosis in Sptlc2^-/-^ DC2.4 cells. Supplementing the media with GM1 partially restores CtxB staining in Sptlc2-/- DC2.4 cells (P<0.05; Fig 9B and 9C), showing successful incorporation of this ganglioside into the membrane. Exogenous addition of GM1 to Sptlc2^-/-^ DC2.4 cells also partially restores phagocytosis of *C*. *albicans* (P<0.05; Fig 9E). Our data suggest a role for GM1 as one of the gangliosides that contributes to phagocytosis.

## Discussion

The *de novo* biosynthesis of sphingolipids starts in the ER. Further conversion to higher sphingolipids takes place at the Golgi. These lipids then accumulate primarily at the outer leaflet of the plasma membrane [17]. Sphingolipids thus serve as a possible site of first contact for incoming pathogens during phagocytosis. Sphingolipids and their metabolites contribute to a variety of cellular processes, including apoptosis, cell growth and membrane transport [52–54]. The role of phosphoinositides in phagocytosis, including phosphatidylinositol 4,5-bisphosphate (PI(4,5)P_2_) and PI(3,4,5)P_3_ is a matter of record [9–14]. The gram-negative bacteria *Neisseria gonorrhoeae* exploits acid sphingomyelinase activity during its opsonization-independent uptake by phagocytes [55]. On the other hand, inhibition of sphingolipids biosynthesis using fumonisin B1 enhances phagocytosis of opsonized red blood cells [20]. Far less is known about the significance of sphingolipids or their metabolites in phagocytosis of fungal pathogens, such as *C*. *albicans*.

We present six lines of evidence to show that sphingolipids are essential for phagocytosis of *C*. *albicans*: (i) myriocin-mediated inhibition of SPT impairs phagocytosis of *C*. *albicans* by macrophages and dendritic cells; (ii) inhibition by FB1 of ceramide synthase, another crucial enzyme in the sphingolipid pathway, results in a stark reduction of *C*. *albicans* phagocytosis; (iii) Sptlc2-deficient dendritic cells, generated through CRISPR/Cas9 mediated genome editing, are defective in phagocytosis of *C*. *albicans;* (iv) exogenous addition of the ganglioside GM1 partially restores the defect in phagocytosis in Sptlc2^-/-^ DC2.4 cells; (v) administration of FB1 *in vivo* sensitizes mice to *C*. *albicans* infection. This corroborates our *in vitro* observations and underscores the importance of sphingolipid homeostasis in clearing fungal infections; (vi) lipidomic analysis is entirely consistent with the specificity of the inhibitors used and with the genetic defect in Sptlc2^-/-^ DC2.4 cells. Of note, sphingolipids levels-normally ~10% of total lipids- are strongly reduced, but residual sphingolipids remain, produced via salvage pathways [34, 35] or possibly acquired from tissue culture media.

Why is sphingolipid biosynthesis critical for phagocytosis, and what is the step at which this class of lipids exerts its most pronounced effect? Phagocytosis is a complex process that involves (i) particle recognition through interaction of pattern recognition receptors (PRRs) on the surface of the phagocyte with ligands on the surface of the particle; (ii) assembly of actin and its associated proteins at the site of ingestion, and formation of a phagocytic cup; (iii) disassembly of actin at the phagosome, and (iv) maturation of the phagosome [3, 42, 56]. Our data show that Sptlc2-deficient cells are defective in the binding stage of phagocytosis.

Sptlc2^-/-^ DC2.4 cells do not form a typical phagocytic cup to engulf particulates. Phagocytosis is an actin-driven process, where sphingolipids may facilitate formation of actin-rich pseudopods generated through membrane curvature at the site of engulfment. The seemingly random polymerization of actin in Sptlc2^-/-^ DC2.4 cells fails to further guide and shape the membrane around the particulate to complete formation of a phagocytic cup. Actin-modulating factors, including the phosphoinositides PI(4,5)P_2_ and PI(3,4,5)P_3,_ may not be recruited properly in Sptlc2^-/-^ DC2.4 cells [57, 58]. The lack of sphingolipids at this site may also alter expression levels and affect lateral mobility of receptors or co-factors critical for particulate recognition. Consistent with this notion, surface display of PRRs such as Dectin-1, TLR2 and FcγR is reduced when sphingolipid biosynthesis is compromised.

Sphingolipids are involved in protein transport from the trans-Golgi network (TGN) to the cell surface [28, 59]. The formation of protein carrier vesicles that bud from the TGN to deliver their cargo to the cell surface requires sphingolipids [28, 59], and perturbation of sphingolipid biosynthesis might therefore affect cargo sorting. Sphingolipids have affinity for cholesterol and form lipid microdomains with distinct protein composition [51]. Depletion of cholesterol-far more abundant in molar concentration than sphingolipids- through application of compounds such as methyl-β-cyclodextrin affect trafficking of raft-associated proteins and some transmembrane proteins, including the influenza virus glycoprotein hemagglutinin [28, 51]. Moreover, length and composition of transmembrane (TM) domains are essential for proper membrane sorting and protein insertion at the plasma membrane [60, 61]; sphingolipids may play a role in this aspect as well.

**Fig 10:**
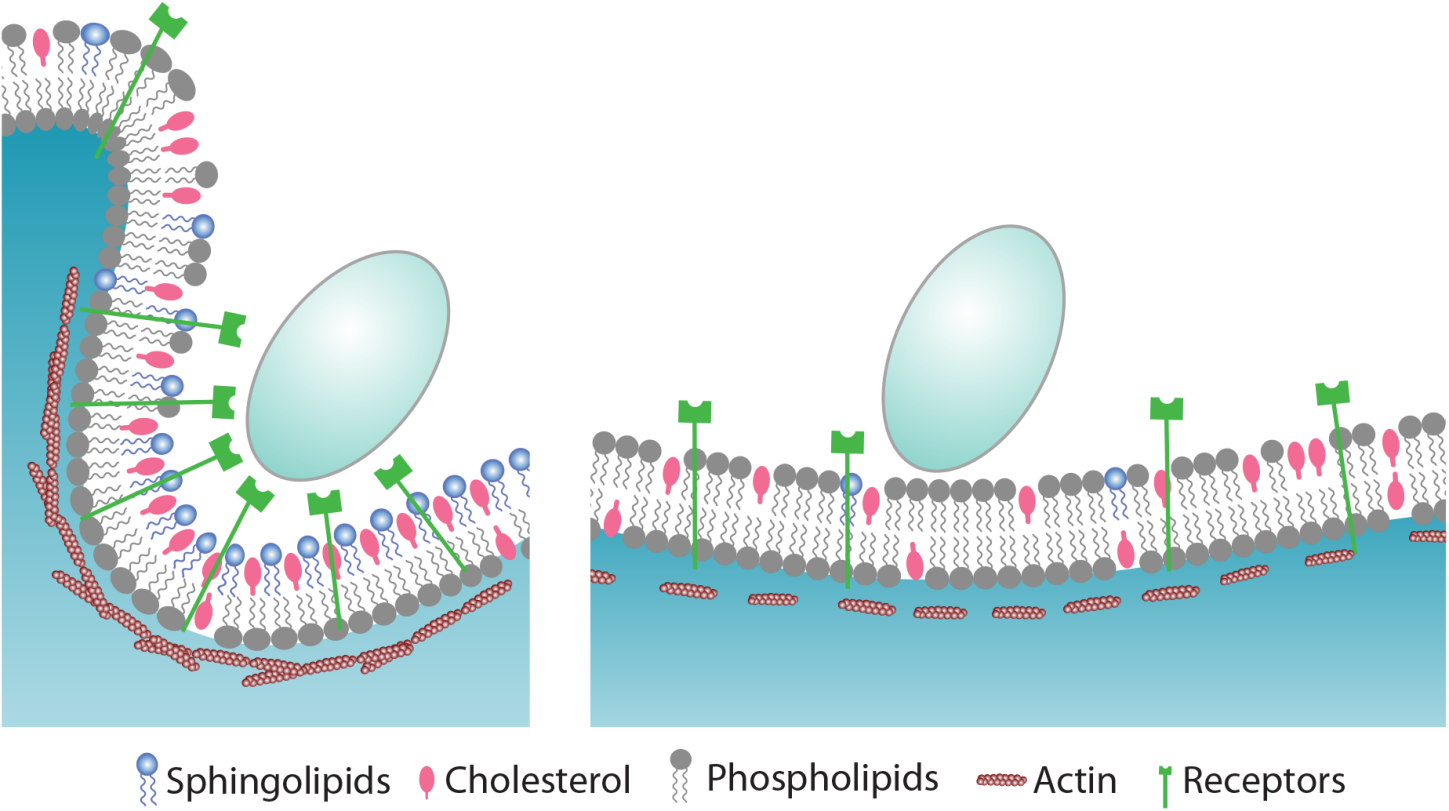
Proposed model for the role of sphingolipids during phagocytosis of *C*. *albicans*. *C*. *albicans* is recognized by pattern recognition receptors (PRRs) that reside at the cell surface of phagocytes. In sphingolipid-depleted cells (left panel), the expression of PRRs such as Dectin 1 and TLR2 is compromised which result in impaired binding of particulate. Sphingolipids accumulate mainly in the outer leaflet of the plasma membrane where they modulate the assembly of actin and its associated proteins to drive phagocytic cup formation. It involves extensive membrane reorganization where sphingolipids contribute, also via trans-bilayer lipid-lipid interactions, to a platform where receptors and signaling molecules such as phosphoinositides transiently accumulate to recruit and activate effector proteins. While sphingolipids and cholesterol cluster around the ingestion site, other phospholipids such as PC and PE are excluded. By virtue of their chemical structure, sphingolipids may facilitate membrane curvature to create the phagocytic cup. and internalization of a fungal particle. See text for details.

Sphingolipids can affect formation of a phagocytic cup in various ways. The lipid composition at the cell surface, where receptors and other regulatory proteins operate, is critical for successful formation of the phagocytic cup. Certain classes of proteins are recruited to the phagocytic cup, whereas others are excluded [62]. Dectin 1-the major *C*. *albicans* receptor that recognizes β-glucans on the cell wall of the fungus- clusters around the synapse-like structures where cup formation is initiated, whereas the regulatory tyrosine phosphatases CD45 and CD148 are excluded from that site [63]. Inevitably, such dynamic movement of proteins would involve extensive membrane reorganization at the plasma membrane in which sphingolipids may play a critical role. Sphingolipids are essential for membrane curvature during intra-luminal vesicle budding [64, 65], membrane remodeling events induced by viruses [66], toxins [67], or plasma membrane damage [68]. Similarly, the actin-rich pseudopods formed through membrane curvature at the site of engulfment are likely facilitated by the presence of sphingolipids (Fig 10).

We find that sphingolipid biosynthesis is dispensable for VSV and vaccinia virus entry, thus demonstrating a specific role for sphingolipids in phagocytosis. While VSV uses clathrin-mediated endocytosis [50], vaccinia virus employs macropinocytosis to enter host cells [49]. Unlike phagocytosis, macropinocytosis- an endocytic mechanism normally involved in fluid uptake- is not accompanied by formation of a recognizable coat and does not require clustering of receptors at the cell surface [69, 70].

FB1-treated mice injected with UV-irradiated *C*. *albicans* showed no signs of disease. When inoculated with viable *C*. *albicans*, FB1-treated mice displayed a higher fungal load in both kidney and brain than control mice, consistent with an inability of the treated mice to clear the fungus through phagocytosis. This further suggests that the cause of death in these mice was systemic candidiasis as a result of a defect in phagocytosis, rather than septic shock resulting from intravenous exposure to TLR ligands. The exact mechanism that underlies this accelerated death remains to be determined. Our observation that Sptlc2^-/-^ DC2.4 cells produce reduced levels of the pro-inflammatory cytokine indicates involvement of sphingolipids in the innate immune response.

Sphingolipids influence the order of the lipid phase at the plasma membrane [71]. Our finding that shows Sptlc2^-/-^ DC2.4 cells have a significantly reduced level of CtxB staining, the lipid raft marker, is consistent with this notion. Specializations of the eukaryotic plasma membrane include lipid domains enriched in (glyco)sphingolipids and cholesterol, often referred to as lipid rafts [72]. The physical characterization of these structures suggests a dynamic nature, with no agreement on either their actual size or life span in the living cell at physiological temperatures [73, 74]. These specializations may serve to organize the disposition of signal transduction cascades through recruitment of key components [28]. In earlier work we have explored in detail the internalization of the *C*. *albicans* and analyzed the contribution of signaling platforms that include Bruton's tyrosine kinase (Btk) and Vav1, in addition to the well-established role of Syk as a downstream kinase important for the function of the C-type lectin dectin-1 [75, 76]. Enzymes such as Btk and Vav1 are amongst the candidates recruited to cytoplasmic lipid-based signaling platforms. Many of the receptors served by Syk, Btk and Vav1 take up residence in sphingolipid-rich membrane compartments to coordinate cascades of signaling events [77].

In nature, dysregulation of sphingolipid biosynthesis is linked to various diseases. Genome-wide association studies link ORM-Like protein isoform 3 (ORMDL3) a member of the ORM gene family, to the onset of childhood asthma [78]. Orm proteins negatively regulate sphingolipid biosynthesis by acting as homeostatic regulators of serine palmitoyltransferase (SPT), the first and rate-limiting enzyme in sphingolipid biosynthesis [79]. Mutation in the Sptlc1 gene that encodes one of the SPT subunits, causes the autoimmune disease hereditary sensory neuropathy type 1 [80]. There are also rare genetic disorders caused by mutations in proteins involved in sphingolipid metabolism [81]. Of note, none of these studies examined the properties of phagocytes.

Collectively, our data show that sphingolipid biosynthesis is essential for clearance of fungal infection through phagocytosis, and hence indispensable for a proper functioning of the innate immune system. Further studies may hold the key to understanding the role of this class of lipids in the coordination of events necessary not only for the removal of such opportunistic fungi, but also for other pathogens, including the many bacteria that exploit the phagocytic pathway to subvert the host’s defense mechanisms.

## Materials and Methods

### Ethics statement

Animals were maintained at the Whitehead Institute for Biomedical Research that is certified by the United States Office of Laboratory Animal Welfare (OLAW) under the guidance of the Public Health Service (PHS) Policy on Humane Care and Use of Laboratory Animals. Whitehead Institute’s Animal Welfare Assurance was approved 11/3/2009 (IACUC, A3125-01). All studies were carried out in accordance with procedures approved by the Massachusetts Institute of Technology Committee on Animal Care (CAC# 1011-123-14).

### Antibodies and chemicals

Rabbit anti- Sptlc2 was purchased from Thermo Scientific Company. All flourophore-conjugated antibodies were purchased from Life Technology. Secondary anti-mouse HRP conjugated antibody was from Sigma Aldrich. Anti-HA-HRP was from Roche. Myriocin and fumonisin B1 was purchased from Sigma Aldrich and Cayman Chemical respectively. 3-indolepropionic acid (IPA-3) and bafilomycin A1 were purchased from Sigma Aldrich.

### Cell cultures

DC2.4 and RAW264.7 cell lines were grown in RPMI medium supplemented with 10% Inactivated Fetal calf Serum (IFS) at 37C and 5% CO_2_. For production of retro- and lenti-viruses, low passage HEK293T cells were transfected using lipofectamine 2000 and virus-containing supernatant was harvested after 48 hours.

### Generation of stable cell lines

pTK93_Lifeact-mCherry was kindly provided by Dr. lain M. Cheeseman lab (Whitehead Institute for Biomedical Research). Cells stably overexpressing pTK93_Lifeact-mCherry were generated by retroviral transduction and subsequent FACS sorting was performed to enrich for mCherry-positive cells.

### Generation of CRISPR/Cas9-mediated Sptlc2^-/-^ DC2.4 cell line

Potential target sequences for CRISPR interference were found with the rules outlined in [82]. The following seed sequences (CRISPR target sequences) preceding the PAM motif that were found in the exon of Sptlc2 gene were used: Sptlc2 #1 GAACGGCTGCGTCAAGAAC; Sptlc2 #2: AGCAGCACCGCCACCGTCG

Potential off-target effects of the seed sequence were confirmed using the NCBI *Mus musculus* Nucleotide BLAST.

Generation of CRISPR/Cas9-mediated Sptlc2-knockout DC2.4 cell line was performed as described in [83]. Briefly, CRISPR gBlock was designed to clone into the restriction enzymatic site NheI/BamHI of CMV promoter-deleted pCDH-EF1-Hygro vector (re-named pCDH-CMV(-)) (SBI; CD515B-1) as follows:

cacagtcagacagtgactcaGTGTCACAgctagcTTTCCCATGATTCCTTCATATTTGCATATACGATACAAGGCTGTTAGAGAGATAATTAGAATTAATTTGACTGTAAACACAAAGATATTAGTACAAAATACGTGACGTAGAAAGTAATAATTTCTTGGGTAGTTTGCAGTTTTAAAATTATGTTTTAAAATGGACTATCATATGCTTACCGTAACTTGAAAGTATTTCGATTTCTTGGCTTTATATATCTTGTGGAAAGGACGAAACACCGnnnnnnnnnnnnnnnnnnnGTTTTAGAGCTAGAAATAGCAAGTTAAAATAAGGCTAGTCCGTTATCAACTTGAAAAAGTGGCACCGAGTCGGTGCTTTTTTTggatccTGTGCACAgtcagtcacagtcagtctac (n: CRISPR target sequences)

The gBlock was then digested using the restriction enzymes NheI and BamHI and ligated into pCDH-CMV(-) vector that was linearized by digesting with the same restriction enzyme.

Doxycycline inducible Cas9 expressing plasmid, pCW-Cas9, was kindly provided by David Sabatini (Whitehead Institute for Biomedical Research). Lentiviruses containing pCW-Cas9 or pCDH-EF1-Hygro-sgRNA were generated as described above. DC2.4 cells were infected with Cas9 lentivirus expressing Cas9 cDNA, and were cultured in media containing 7 μg/mL of puromycin (Sigma Aldrich). These Cas9-inducible cells were re-infected with lentivirus carrying pCDH-CMV(-)-sgRNA, and were cultured in media containing 250 μg/mL of hygromycin B (Life Technology). The cells stably expressing the sgRNA and Cas9 proteins were treated with 2 μg/mL of doxycycline (Clontech) for 3–5 days. The cells were re-plated to a 96-well plate at a density of 0.5 cells per well. The individual colonies were collected and the expression of Sptlc2 was examined by western-blotting using Sptlc2 antibody.

### Myriocin and fumonisin B1 treatment of cell lines

The cells were either treated with 0.5μg/ml myriocin or 0.25μg/ml FB1 for 4 days and, after which cells were infected with *Candida*-BFP for phagocytosis experiments. Control cells were treated with only DMSO/PBS. Reduction in production of sphoingolipid biosynthesis in myriocyn/FB1 treated cells was verified by metabolic labeling and lipid analysis by TLC.

### Metabolic labeling and TLC analysis of lipids

Around 2 million cells were grown in 6-well-dishes and labeled with 3 μCi/mL of N-methyl-[^14^C]-choline or 3-L-[^14^C]-serine for 5 hours in Opti-MEM at 37°C. Cells were washed two times with PBS and lipid extraction was done following Bligh and Dyer method [84]. The methanol/chloroform-lipid extracts were dried by nitrogen gas. Dried lipids were re-dissolved in a few drops of chloroform/methanol (1:2, vol/vol) and loaded on a TLC plate. Where indicated, glycerolipids were removed by mild alkaline hydrolysis in 0.5 M sodium methoxide in MeOH for 1 h at RT. Lipids were separated by developing the TLC plate first in acetone and then in chloroform, methanol, 25% ammonia solution (50:25:6, vol/vol/vol). Radiolabeled lipids were detected on a Phosphor-Imager (Fujifilm BAS-2500) using Image Reader BAS-2500 V1.8 (Fujifilm).

### Lipid mass spectrometry analysis of liver tissue and cell lines

Lipids were extracted from DC2.4 cell lines and liver tissue of mice treated or untreated with FB1 according to Folch et al. [85] but without the salt. Lipid extracts were then separated on an Ascentis Express C18 2.1 x 150 mm 2.7 μm column (Sigma-Aldrich, St. Louis, MO) connected to a Dionex UltiMate 3000 UPLC system and a QExactive benchtop orbitrap mass spectrometer (Thermo Fisher Scientific, San Jose, CA) equipped with a heated electrospray ionization (HESI) probe. Dried lipid samples were typically dissolved in 50 ul 65:30:5 acetonitrile:isopropanol:water (v/v/v) and 5 ul was injected into the LC/MS, with separate injections for positive and negative ionization modes. Mobile phase A in the chromatographic method consisted of 60:40 water/ACN in 10 mM ammonium formate and 0.1% formic acid, and mobile phase B consisted of 90:10 IPA/ACN, also with 10 mM ammonium formate and 0.1% formic acid. The chromatographic gradient was described previously [86]. The column oven and autosampler tray were held at 55°C and 4°C, respectively. The MS instrument parameters were as described previously [87]. The spray voltage was set to 4.2 kV, and the heated capillary and the HESI were held at 320°C and 300°C, respectively. The S-lens RF level was set to 50, and the sheath and auxiliary gas were set to 35 and 3 units, respectively. These conditions were held constant for both positive and negative ionization mode acquisitions. External mass calibration was performed using the standard calibration mixture every 7 days.

MS spectra of lipids were acquired in full-scan / data-dependent MS^2^ mode. For the full scan acquisition, the resolution was set to 70,000, the AGC target was 1e6, the maximum integration time was 50 msec, and the scan range was m/z = 133.4–2000. For data-dependent MS^2^, the top 10 ions in each full scan were isolated with a 1.0 Da window, fragmented at a stepped normalized collision energy of 15, 25, and 35 units, and analyzed at a resolution of 17,500 with an AGC target of 2e5 and a maximum integration time of 100 msec. The underfill ratio was set to 0. The selection of the top 10 ions was subject to isotopic exclusion, a dynamic exclusion window of 5.0 sec, and an exclusion list of background ions based on a solvent blank.

High-throughput profiling of lipidomic data was performed using LipidSearch software (Thermo Fisher Scientific / Mitsui Knowledge Industries) [88, 89]. In addition, sphingosine and sphinganine were manually analyzed and matched to reference spectra (http://metlin.scripps.edu) using XCalibur QualBrowser software, and peaks were quantified by XCalibur QuanBrowser software (Thermo Fisher Scientific).

### Phagocytosis assays

*C*. *albicans* strain SC5314 was cultured in YPD + Uri (2% bactopeptone, 1% yeast extract, 2% glucose and 80 μg/ml Uridine) at 37°C. Generation of *Candida albicans* expressing blue fluorescent protein (*Candida*-BFP) is described in [75]. Zymosan A was labeled with Alexa647 carboxylic acid (succinimidyl ester) by incubation in 0.1 M Na_2_CO_3_ at room temperature. For immunofluorescence microscopy, *Candida*-BFP (at an MOI of 10), zymosan-Alexa647 or latex beads-rabbit IgG-FITC were added (10 particles per cell) to the cells that were plated on a cover slip a day earlier. Typically, cells were fixed after 60 and 90 min post infection. The time points selected are based on our previous studies [75]. *C*. *albicans* begins to form hyphae at ~60 min post infection; phagocytes begin to show cell death (pyroptosis) after ~90 min. At different time points, cells were washed twice with PBS and fixed with 4% PFA for 30 min at room temperature. Cells were washed with PBS and incubated in 50mM NH_4_CL in PBS for 10 min and incubated for another 30 min with binding buffer (0.1% saponin, 0.2% BSA in PBS). Cells were stained with phalloidin-Alexa 488 or 568 (Life Technology) for 60 min and washed several times with PBS and mounted on slides for confocal microscopy.

Peritoneal macrophages were harvested by peritoneal lavage with upto 10ml PBS. Cells were seeded on coverslips in DMEM (high glucose; Gibco) with 10% FCS plus 0.5μg/ml myriocin and used for phagocytosis experiments four days later.

### Microscopy

All images were captured in the W.M. Keck facility for Biological Imaging using a PerkinElmer live cell imaging spinning disk confocal system and Volocity software.

Images were captured using a confocal microscope with a 63°- 1.40 N.A. of the Carl Zeiss Plan Apo oil objective. ImageJ was used to quantify the fluorescent intensity of the images.

### FB1 treatment of mice and *C*. *albicans* infection

Animals were housed at the Whitehead Institute for Biomedical Research and maintained according to protocols approved by the Massachusetts Institute of Technology Committee on Animal Care. C57BL/6 wild type mice were purchased from Jackson Labs. C57BL/6 mice were treated daily with 2mg/kg FB1 subcutaneously for five days. Control and FB1 treated animals (10 mice per group) were left uninfected (received only PBS) or infected with live or UV killed 2x 10^4^ CFU of *C*. *albicans* in PBS via tail vein injection The mice continued to receive FB1 daily for the rest of the study, during which the health and overall well-being of the animals was monitored. Ten mice per group were used, and three independent experiments were performed. For the analysis of fungal load in the kidney and brain, the animals were sacrificed at the late stage of the disease (9 days after injection of *C*. *albicans*). To study the fungal loads, brain and kidney tissues of the whole organs were homogenized in PBS. Serial dilutions of the homogenates were grown on YPD plates and colonies were counted after 3 days.

### Virus production

VACV WR E eGFP [48] encoding eGFP under the control of the J2R early (E) promoter in the tk locus was produced in BSC-40 cells and purified from cytoplasmic extracts through a 16% sucrose cushion in 20 mM Tris pH 9.0. VSV GFP was produced in Vero cells and virus-containing cell supernatants used for infections [48].

### Flow cytometry-based virus infection assays

To quantify infection by flow cytometry, DC2.4 wt or Sptlc2 knockout cells were seeded in 24-well plates one day before infection (2·10^5^ cells/well). Cells were infected with the appropriate amounts of WR E eGFP or VSV GFP (in DMEM). 30 minutes post infection, inoccula were removed and cells cultivated for 5:30h in full medium. Cells were trypsinized and fixed in 4% formaldehyde/PBS. Green fluorescent cells were quantified using a BD Biosciences LSRFortessa flow cytometer and the FlowJo software package.

### Pulse chase and analysis of cell supernatants

About 10 × 10^6^ wild type and Sptlc2^-/-^ DC2.4 cells were starved in methionine- and cysteine-free DMEM and starved for 30 min at 37°C. Cells were pulse-labelled for 20 min with [^35^S]-methionine/cysteine at 0.77 mCi/mL. Cells were then chased in complete media for 0, 30, and 90 min. At different time points during the chase cells were collected, washed once with cold PBS, and lysed in Tris buffer [150 mM NaCl, 5 mM MgCl_2_, 25 mM Tris (pH 7.4)] containing 0.5% Nonidet P-40. The lysates were precleared with immobilized protein G beads for 3 h, and MHC class I molecules were recovered using an H-2K^b^ heavy chain specific (p8) rabbit serum [90]. Immunoprecipitated samples were subjected to Endo H treatment according to the manufacturer’s instructions. For analyzing the secretome, cells were labeled as described above and chased for the indicated time points in a media without serum. Supernatants were collected, boiled in SDS sample buffer and analysed by SDS/PAGE. Samples were visualized with autoradiography using DMSO/PPO (2,5-diphenyloxazole) and exposure to Kodak XAR-5 film.

### Flow cytometry analysis to measure cell surface expressions

Equal numbers (about 0.5x10^6^) of control and Sptlc^-/-^ DC2.4 cells were incubated with antibodies for 20 min on ice. The cells were washed with cold PBS supplemented with 1% BSA and subjected to cytofluorometry immediately (FACSCalibur; BD Biosciences). Control samples were prepared using the corresponding isotype antibody. FlowJo was used to analyze the data. Intensity of fluorescence was measured, and the percent maximum presented in the overlaid histograms, and mean fluorescence intensity (MFI) was measured to calculate the standard deviation between the experiments.

## Supporting Information

S1 Fig The significance of time-course blockade of sphingolipid biosynthesis using myriocin on cellular lipidome and phagocytosis of *C*. *albicans*. (A-E) DC2.4 cells were treated with myriocin for different time points (0, 1, 2, 3 or 4 days). Total lipids were extracted from untreated (time 0) or treated cells and lipid profiling was performed using LC/MS. Levels of cerebrosides (A), ceramide (B), sphingomyelin (C), sphinganine (D), and phosphatidylcholine (E) are shown. (F) Myriocin treated and untreated DC2.4 cells were infected with C*andida*-BFP for 60 min. Cells were fixed and the internalized C*andida*-BFP was examined by confocal microscopy. The internalized *Candida*-BFP were quantified and presented as the percentage relative to the control. All graphs display SD of three independent experiments, and an unpaired t-test was used to analyze the significance of the data. ** p < 0.001, * p < 0.05.
(EPS)

S2 Fig Sptlc2^-/-^ DC2.4 cells have normal morphology and cell division. (A) Electron microscopy images showing the overall structure of control and Sptlc2^-/-^ DC2.4 cells. (B) Similar number of the control and Sptlc2^-/-^ DC2.4 cells were seeded in a 6-well dishes. Quantification of the number of live cells after 24 h and 48 h is shown.
(EPS)

S3 Fig Sptlc2^-/-^ DC2.4 cells display impaired phagocytosis of zymosan and IgG-coated latex beads. Confocal images of wild type and Sptlc2-deficient DC2.4 cells incubated for 30 min with zymosan-Alexa647 (A) or latex beads-rabbit IgG-FITC (B). Cells were fixed and stained with phalloidin-Alexa 488 to visualize the outline of the cells. (C–F) Quantification of the number of internalized zymosan (C) or IgG-beads (E), and the number of zymosan (D) or IgG-beads (F) per cell is shown. Experiments were done as in A and B. The internalized particulates were quantified and presented as the percentage relative to the control. Error bars display SD of three independent experiments, and unpaired t-test was used to analyze the significance of the data. ** p < 0.001.
(EPS)

S4 Fig The effect of FB1 on mice body weight and growth of *C*. *albicans*. (A) Mice received daily subcutaneous injections of 2 mg/kg FB1 or the vehicle (PBS), and their body weight at different time points is shown. (B) *C*. *albicans* were grown in liquid culture media supplemented with 25μg/ml. The growth of the *C*. *albicans* at different time points was measured using spectrometry (OD_600_) and presented as percent relative to the control.
(EPS)

S5 Fig Sptlc2^-/-^ DC2.4 cells do not show a generic defect in membrane trafficking. (A) Equal numbers of wild type and Sptlc2^-/-^ DC2.4 cells were pulse-labeled with [^35^S]-methionine/cysteine for 30 min and chased for different time points at 37°C or kept on ice. Supernatants were collected and analyzed by SDS/PAGE and autoradiography. (B) Cells that were pulse-labelled like in A were collected and total cell lysates were analysed by SDS/PAGE and autoradiography.
(EPS)

S1 Movie A movie showing a z-stack confocal microscopy image of DC2.4 cells that internalized *C*. *albicans* (shown in red). Cells were stained with Alexa488-conjugated phalloidin to visualize cell contour.
(AVI)

S2 Movie A movie showing live confocal microscopy imaging of the formation of a phagocytic cup upon binding of *C*. *albicans* (shown in red) in control DC2.4 cells that stably express LifeAct (shown in green). (AVI)

S3 Movie A movie showing live confocal microscopy imaging of the formation of a phagocytic cup upon binding of *C*. *albicans* (shown in red) in SPT2-deficient DC2.4 cells that stably express LifeAct (shown in green). (AVI)
